# Testing the association between the enabling and enacting factors of patient safety culture and patient safety: structural equation modelling

**DOI:** 10.1186/s12912-023-01196-x

**Published:** 2023-02-06

**Authors:** Seung Eun Lee, V. Susan Dahinten, Jong Hyun Lee

**Affiliations:** 1 Mo-Im Kim Nursing Research Institute, College of Nursing, Yonsei University, 50-1 Yonsei-ro, Seodaemun-gu, Seoul, 03722 South Korea; 2 School of Nursing, University of British Columbia, T201–2211 Wesbrook Mall, Vancouver, BC V6T 2B5 Canada; 3 Department of Psychology, College of Social Science, Gyeongsang National University, 501, Jinju-daero, Jinju-si, Gyeongsangnam-do 52828 South Korea

**Keywords:** Safety culture, Safety climate, Structural equation model, Survey, Nurses, Korea

## Abstract

**Background:**

Despite evidence linking a safety culture with patient safety, the processes by which aspect of safety culture influences patient safety are not yet well understood. Thus, this study aimed to test a theoretical model of the relationships between three enabling factors (supervisor/clinical leader support for patient safety, hospital management support for patient safety, and psychological safety), and four enacting factors of patient safety culture (handoffs and information exchange, teamwork, error reporting intention, and withholding voice) with nurse assessments of patient safety.

**Methods:**

A cross-sectional, descriptive correlational study design was used. Between May and June 2020, 526 nurses who provided direct care to patients in medical surgical units in three Korean hospitals completed an online survey that included four standardized scales or subscales. Structural equation modelling was used to test the hypothesized model.

**Results:**

Among the three enabling factors, psychological safety was associated with all four enacting factors, and all enacting factors were associated with overall patient safety. Hospital management support was associated with all enacting factors except teamwork, but supervisor/clinical leader support was associated with only handoffs and information exchange, and withholding voice. Thus, teamwork was influenced only by psychological safety. Findings demonstrate overall support for the theoretical model of safety culture wherein enabling factors influence enacting factors which, in turn, lead to patient safety outcomes, but emphasize the critical nature of psychological safety among nursing staff.

**Conclusion:**

This study provides further insight into the importance of support from hospital management and unit supervisors/clinical leaders for patient safety to motivate and enable hospital nurses to enact behaviours necessary for patient safety. However, such support must also take the form of enhancing psychological safety for nursing staff.

## Introduction

Patient safety remains a priority in healthcare systems around the world, and a weak safety culture has been identified as a critical factor contributing to unsafe patient care [1]. Safety culture, an aspect of organizational culture, is commonly understood as the relatively stable shared beliefs, values, and norms regarding safety within an organization [2, 3]. The underlying premise is that safety culture affects what health care providers perceive as appropriate behaviour in regard to patient safety, and encourages them to engage in those behaviours which, in turn, impact patient safety [4]. In spite of evidence linking a safety culture with patient safety, the studies have tended to be atheoretical, research findings have been mixed, and the processes by which aspect of safety culture influences patient safety are not yet well understood [2, 5]. Thus, it is needed to investigate the impacts of various dimensions of safety culture on nurse-perceived patient safety employing an innovative theoretical model informed by the work of Vogus, Singer and colleagues [6, 7] and Bisbey et al. [2].

Most studies on safety culture draw on measures of safety culture that provide aggregated scores for various dimensions or factors that are hypothesized to comprise safety culture, but there has been a lack of a unifying framework to understand the relationship between such dimensions or how safety culture develops and contributes to patient safety [2]. To address this gap, Vogus and colleagues drew on management research to develop a theoretical model of safety culture that categorizes various activities within the organization as enabling, enacting, and elaborating processes [6, 7]. Leader actions that support patient safety activities by staff were identified as enabling processes. Actions by front line staff to enhance patient safety (e.g., interpersonal processes such as teamwork and information exchange) were identified as enacting processes. Elaborating processes consisted of activities that contribute to organizational learning and the maintenance of a safety culture (e.g., feedback on errors).

A decade later, Bisbey and colleagues [2] drew on the work by Singer, Vogus and colleagues [6, 7] and a further review of the safety culture literature from various industries to develop a framework for understanding how safety culture develops and is sustained in healthcare organizations. Although they asserted that the framework was “not intended to be tested as a theoretical model” (p. 106) the framework does include pathways from enabling factors to safety culture to enacting behaviours to safety outcomes, and finally, a feedback loop from safety outcomes to safety culture. One difference between the framework by Bisbey et al. [2] and the theoretical model by Singer and Vogus [7] is that the latter proposed pathways from enabling factors to enacting factors to safety culture (i.e., safety culture developed as a result of both enabling and enacting factors).

### Enabling factors

Both teams of researchers, Vogus et al. [6] and Bisbey et al. [2], identified organizational or management level activities as a key component of enabling factors. These include leader actions that draw attention to and prioritize the importance of safety, and provide support for safety-related behaviours by staff. Both models also include the notion of psychological safety as an enabling factor, although, whereas Vogus and colleagues identified this as management actions that make it safe for staff to speak up, Bisbey et al. labelled psychological safety more explicitly and defined it as a collective perception that the group was safe for risk-taking. Psychological safety has been identified as an antecedent to error reporting in health care settings [8]. Psychological safety has also been found to be inversely related to withholding voice, that is, maintaining silence rather than speaking up for the purpose of enhancing patient safety [9]. Therefore, in our study, we identified three enabling factors: unit supervisor/clinical leader support for patient safety, hospital management support for patient safety, and psychological safety.

### Enacting factors

Enacting factors include actions by front line staff that prevent or respond to threats to patient safety [6] such as teamwork, communication/information exchange, and the reporting of incidents and other patient safety concerns. Bisbey and colleague’s [2] review of the literature revealed consistent support for the associations between teamwork and positive safety outcomes, and communication/information exchange and positive safety outcomes. Much of the research on safety culture has focused on error reporting [2], but a recent study [9] also showed an inverse relationship between withholding voice and perceived patient safety. Thus, in our study, we identified four enacting factors: handoffs and information exchange, teamwork, error reporting, and withholding voice.

### Aims

**Fig. 1:**
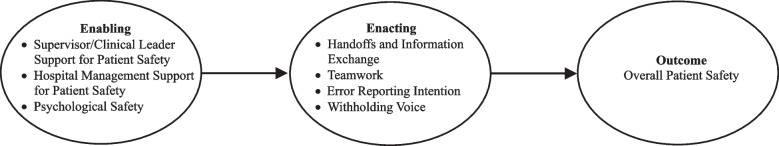
Conceptual model

Much of the previous research on patient safety has been limited by the lack of a theoretical framework, and inattention to the processes or pathways by which safety culture factors may impact patient safety outcomes. Therefore, the aim of this study was to test a theoretical model of the relationships between three enabling factors and four enacting factors of patient safety culture with nurse assessments of overall patient safety. The model being tested in this study proposed that each of the three enabling factors would be associated with each of the four enacting factors which, in turn, would be associated with patient safety (see Fig. 1). All associations were hypothesized to be positive, except for the pathways leading to and from withholding voice.

## Methods

### Study design

A cross-sectional, descriptive correlational study design was used to test the hypothesized relationships between the study variables. The STROBE (Strengthening the Reporting of Observational Studies in Epidemiology) guideline was used to guide this study.

### Participants

This study used data from a convenience sample of nurses who were providing direct care in medical/surgical units, and had at least 6 months nursing experience in their current workplace. The nurses were recruited from three tertiary hospitals that had a minimum total bed capacity of 1000. Nurses who met the inclusion criteria (*N* = 731) were invited to participate in the study via emails sent out by the hospital on behalf of the research team. The invitations included a secure link to an online questionnaire, and the informed consent form was provided on the first page of the questionnaire. A total of 526 nurses completed the questionnaire for a response rate of 72%. The final sample size satisfied the requirements for structural equation modelling (SEM): 200 to 450 participants regardless of the size of the model [10, 11]. More detailed information about sampling is published in another article [9].

### Data collection

To avoid personal contact during data collection in the COVID-19 outbreak, an online survey was carried out. Data were collected between May and June 2020.

### Measures

The survey drew on four standardized scales or subscales to measure the three enabling factors, four enacting factors, and one patient safety outcome. Demographic information (i.e., age, gender, education level, employment status, years of nursing experience, unit tenure, and hospital tenure) was also collected.

Two of the enabling factors, supervisor/clinical leader support for patient safety (3 items) and hospital management support for patient safety (3 items), were measured with subscales from the Korean version of Hospital Survey on Patient Safety Culture (K-HSOPSC 2.0) which has demonstrated acceptable reliability, and content and construct validity [12]. A sample item for supervisor/clinical leader support for patient safety is “My supervisor, manager, or clinical leader seriously considers staff suggestions for improving patient safety.” “Hospital management provides adequate resources to improve patient safety” is an example of an item in the hospital management support for patient safety subscale. Cronbach’s alpha for these scales were 0.75 and 0.72, respectively. Psychological safety was measured using a 7-item scale originally developed by Edmondson [13], and translated into Korean and validated by Lee and Dahinten [9]. A sample item is “It is safe to take a risk on this team.” Cronbach’s alpha for this scale was 0.76. Responses for the three enabling subscales were measured on a 5-point response scale ranging from 1 (strongly disagree) to 5 (strongly agree). A total mean score was computed with higher scores indicating a higher level of each construct. Cronbach’s alpha for this scale was 0.76. Responses for the three enabling subscales were measured on a 5-point response scale ranging from 1 (strongly disagree) to 5 (strongly agree). A total mean score was computed with higher scores indicating a higher level of each construct.

Two enacting factors, handoffs and information exchange (3 items) and teamwork (3 items), were measured with subscales from the K-HSOPSC 2.0 [12]. A sample item for handoffs and information exchange is “During shift changes, important patient information is often left out.” An example item for teamwork is “In this unit, we work together as an effective team.” Responses were measured on a 5-point response scale ranging from 1 (strongly disagree) to 5 (strongly agree). For each subscale, a total mean score was computed with higher scores indicating higher levels of the construct. Cronbach’s alpha was 0.77 for teamwork, and 0.72 for handoffs and information exchange.

Error reporting intention was measured using a 3-item scale developed by Kim [14]. An example item is “If you made an error that did not harm the patient, would you report it?” Responses were measured on a 5-point scale ranging from 1 (never) to 5 (always). A total mean score was computed, with a higher score indicating a higher level of error reporting intention. Cronbach’s alpha for this study was 0.81.

Withholding voice was measured using a 4-item scale originally developed by Richard et al. [15] and translated into Korean and then validated by Lee and Dahinten [9]. The items assessed the frequency of withholding voice in specified situations during the past 4 weeks, and an example item is “Over the past week, did you choose not to bring up your specific concerns about patient safety?” Responses were measured on a 5-point Likert scale ranging from 1 (never, 0 times) to 5 (very often, more than 11 times). A total mean scale score was computed with a higher score indicating a higher level of withholding voice. Cronbach’s alpha for this study was 0.90.

Patient safety was measured with a single-item from the K-HSOPSC 2.0 [12]. Participants were asked to assign their unit an overall rating on patient safety using a 5-point scale ranging from 1 (poor) to 5 (excellent). This item has been used in previous studies on patient safety culture [16–18], and is considered a reliable and valid outcome measure.

### Data analysis

For all measures, we reverse-coded negatively worded items for statistical analyses.

Descriptive statistics were calculated for the demographic characteristics of study participants and key study variables, and bivariate correlation analyses were conducted to examine the relationships between key study variables. The hypothesized relationships between three enabling factors, four enacting behaviours, and patient safety were examined by employing SEM. Structural equation modelling can simultaneously test a series of hypothesized relationships between variables to determine whether or not the data are consistent with the hypothesized model. Before conducting the multivariate analysis, the skewness and kurtosis of each variable were examined and found to be normally distributed as required by SEM [10]. Model fit was evaluated based on the following fit statistics: a value < 3.0 for the normed chi-squared (*χ2*/*df*) (Ma & Zhou, 2020), a value ≥ .9 for the comparative fit index (CFI) and Tucker–Lewis index (TLI), a value < .06 for the root mean square error (RMSEA), and a value < .08 for the standardized root mean residual (SRMR) [10, 19]. SPSS 25.0 and AMOS 24.0 were used for data analysis with a significant level at *α* = .05.

## Results

### Descriptive statistics

Almost all of the 526 participants were female (98%) with a mean age of 31.2 years (*SD* = 11.3). Most (95%) had a baccalaureate or higher degree in nursing and were permanent, full-time employees (99%). The participants’ mean years of nursing experience was 7.5 years (*SD* = 6.5). Their average unit tenure was 4.4 years (*SD* = 3.9) and average hospital tenure was 7.1 years (*SD* = 6.5). Descriptive statistics for the key study variables are presented in Table 1.

**Table 1:** Descriptive Statistics for Key Study Variables (*N* = 526)

Variables	*M*	*SD*
Supervisor/Clinical Leader Support for Patient Safety	3.71	.61
Hospital Management Support for Patient Safety	2.94	.72
Psychological Safety	3.36	.51
Handoffs and Information Exchange	3.39	.63
Teamwork	3.55	.61
Error Reporting Intention	3.59	.70
Withholding Voice	1.76	.69
Patient Safety	3.30	.67

### Bivariate analysis

Table 2 presents inter-correlations between key study variables. The pattern of correlations was consistent with theoretical expectations. Correlations between the enabling and enacting safety culture factors ranged from .15 (*p* < .001) between supervisor/clinical leader support and error reporting intentions to .46 (*p* < .001) between psychological safety and teamwork. Correlations between the enabling safety culture factors and patient safety ranged from .16 (*p* < .001) for error reporting intentions to .33 (*p* < .001) for handoffs and information exchange. As expected, all associations were positive except for those with withholding voice which was inversely correlated with all other theoretical constructs.

**Table 2:** Pearson Correlations between Key Study Variables (*N* = 526)

Variables	1	2	3	4	5	6	7
1. Supervisor/Clinical Leader Support for Patient Safety	_						
2. Hospital Management Support for Patient Safety	.23^***^	_					
3. Psychological Safety	.45^***^	.20^***^	_				
4. Handoffs and Information Exchange	.28^***^	.37^***^	.28^***^	_			
5. Teamwork	.32^***^	.16^***^	.46^***^	.28^***^	_		
6. Error Reporting Intention	.15^***^	.19^***^	.23^***^	.10^*^	.07	_	
7. Withholding Voice	−.28^**^	−.22^**^	−.26^***^	−.22^***^	−.14^**^	−.18^***^	_
8. Patient Safety	.30^***^	.33^***^	.29^***^	.33^***^	.26^***^	.16^***^	−.25^***^

^*^p < .05. ^**^ *p* < .01. ^***^ *p* < .001

### Structural equation modelling

Prior to testing the SEM model, we created a latent variable for the patient safety, which was measured using a single item. Because its construct reliability cannot be calculated, we first calculated the square root of the means of reliability coefficients of all other key study variables. Then, we included the value in the path between the latent variable and the observed variable for patient safety, and the remaining ratio of the reliability (1-α) was assigned to the variance of the error term [20, 21].

The chi-square test of absolute model fit was statistically significant as expected due to the large sample size, χ2/*df* ratio = 2.37 (*χ2* = 724.93 *df* = 306, *p* < .001). The fit indices indicated that the hypothesized model provided an acceptable fit to the data: RMSEA = .05, TLI = .91, SRMR = .06, and CFI = .92.

**Fig. 2:**
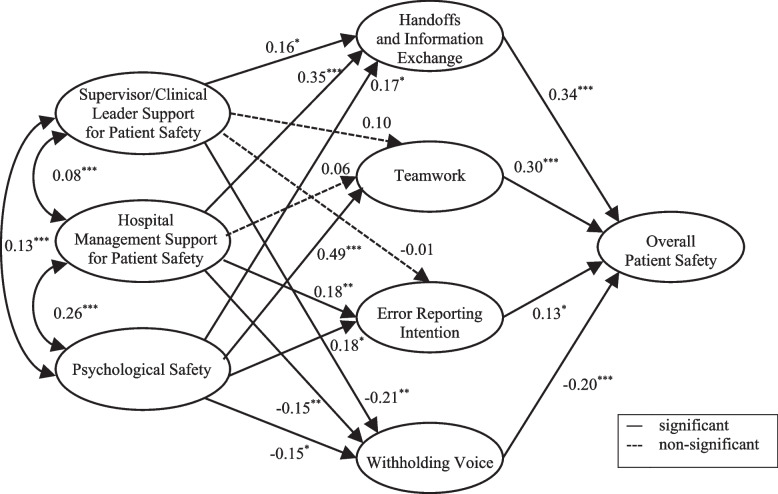
Theoretical model with standardized path coefficients. *Note*. Standardized coefficients are presented. ^*^p < .05. ^**^ *p* < .01. ^***^ *p* < .001

Figure 2 shows the standardized coefficients for pathways between latent constructs.

Among the three enabling factors, only psychological safety was significantly associated with each of the enacting factors: handoffs and information exchange (*β* = .17, *p* = .019), teamwork (*β* = .49, *p* < .001), error reporting intention (*β* = .18, *p* = .014), and withholding voice (*β* = −.15, *p* = .027). Supervisor/clinical leader support was significantly associated with handoffs and information exchange (*β* = .16 *p* = .022) and withholding voice (*β* = −.21, *p* = .002), but not with error reporting intention (*β* = −.01, *p* = .915) or teamwork (*β* = .10, *p* = .151). Hospital management support for patient safety was significantly related to handoffs and information exchange (*β* = .35, *p* < .001), error reporting intention (*β* = .18, *p* = .001), and withholding voice (*β* = −.15, *p* = .004), but not with teamwork (*β* = .06, *p* = .291). As expected, all enacting factors were significantly related to patient safety: (*β* = .34, *p* < .001 for handoffs and information exchange; *β* = .30, *p* < .001 for teamwork; *β* = .13, *p* = .032 for error reporting intention; and *β* = −.20, *p* < .001 for withholding voice). As expected, all the statistically significant pathways were positive except for the pathways leading to and from withholding voice.

Additional analyses were conducted to examine the total indirect effect from each enabling factor to patient safety through the four enacting factors. Bias-corrected bootstrap (1000 samples) with a 95% confidence interval was used to obtain stable and valid standard errors of the estimates [22]. As shown in Table 3, all enabling factors appeared to have a significant indirect influence on patient safety through each enacting factor.

**Table 3:** Standardized Total Indirect Effects and 95% Confidence Intervals

Path			*Estimate*	*SE*	95% CI Bias-Corrected
Supervisor/Clinical Leader Support for Patient Safety	→	Patient Safety	.13	.05	(.04, .22)
Hospital Management Support for Patient Safety	→	Patient Safety	.19	.04	(.10, .28)
Psychological Safety	→	Patient Safety	.26	.06	(.15, .37)

This study found that all enabling factors were significantly and positively associated with handoffs and information exchange, and handoffs and information exchange was, in turn, significantly and positively related to patient safety. Among all enabling factors, only psychological safety had a significant and positive relationship with teamwork, and teamwork was, in turn, positively associated with patient safety. Among the three enabling factors, only hospital management support for patient safety and psychological safety were significantly and positively related to error reporting intention, and error reporting intention was, in turn, positively associated with patient safety.

## Discussion

The purpose of this study was to test a theoretical model of the relationships between three enabling factors and four enacting factors of patient safety culture with nurse assessments of patient safety. We hypothesized that each of the enabling factors (supervisor/clinical leader support for patient safety, hospital management support for patient safety, and psychological safety) would be associated with each of the enacting factors (handoffs and information exchange, teamwork, error reporting intention, and withholding voice) which, in turn, would be associated with patient safety. Our findings add to a limited body of work addressing how various aspects of safety culture interact to influence safety outcomes in healthcare organizations.

The most notable finding of this study was that among the three enabling factors, only psychological safety was associated with all four enacting factors. Previous empirical research [9] and a literature review of other studies [23] have also shown associations between psychological safety and teamwork, error reporting, and withholding voice in healthcare settings. When nurses feel psychologically safe to take risks, they are more likely to share important information for patient safety, actively engage in teamwork behaviours, and report errors and near misses. Also, when nurses feel psychologically safe, they are less likely to withhold their voices when they have concerns about patient safety or ideas for improvements in patient care. These behaviours would, in turn, have positive impacts on patient safety in the unit. Similar to our findings, psychological safety was positively associated with nurses’ speaking up behaviours regarding patient safety in Dutch hospitals [24]. However, in the Dutch study, the outcome was speaking up rather than withholding voice (i.e., silence). Different researchers hold different views on whether speaking up and withholding voice are distinct concepts or opposite ends of a continuum [25, 26].

Nurse leaders at the unit level and hospital administrators have an important role to play in facilitating psychological safety. At the unit level, nurse managers should demonstrate inclusiveness by seeking out opposing viewpoints, showing a willingness to listen and respond to staff concerns and recommendations, showing appreciation for staff input, and treating the staff with respect [9, 27]. Nurse leaders could also model risk taking by admitting faults. Hospital management could contribute by providing team-based training on professional communication and interpersonal risk taking. Each staff member should understand and develop skills in interpersonal risk-taking, but these skills will take hold best when practiced in teams [28].

As hypothesized, when nurses perceived higher levels of hospital management support for patient safety, they reported higher levels of information exchange and error reporting intentions, and less frequently withheld their voices for patient safety. When hospital managers express their commitment to safety, nurses may use them as role models for determining the behaviours that are expected within the organization [2]. In addition to role modeling the patient safety values of the organization, hospital management may provide support for patient safety by establishing policies regarding incident reports and a non-punitive environment for reporting errors. In addition, hospital management should strive to create an environment where speaking up is not only acceptable but expected as a professional responsibility. This might include establishing an appropriate reward system to encourage staff to contribute their ideas for patient safety.

Contrary to expectations, our multivariate analyses did not show an association between hospital management support for patient safety and unit teamwork. This might be because all study participants worked at tertiary hospitals with a minimum bed capacity of 1000. In such large healthcare organizations, the impact of hospital management support on unit teamwork might be limited due to the distance between staff nurses and hospital management (i.e., staff nurses would have little direct interaction with hospital management). Thus, it might be expected that teamwork would be more strongly influenced by unit supervisors and clinical leaders [29]; however, this relationship was also not supported by the structural equation modelling results. Teamwork did show associations with both hospital management and supervisor/clinical leader support for patient safety in the bivariate analyses, but not in the multivariate analyses. Thus, another possibility is that the influence of both of these enabling factors on teamwork might be mediated through another variable, such as psychological safety. Thus, further research is needed to better understand the processes and pathways through which enabling factors influence enacting factors.

In line with the theoretical framework of Bisbey and colleagues [2], the current study demonstrated that all enacting factors have effects on patient safety in healthcare organizations, and that all enabling factors have indirect effects on patient safety. Additionally, our findings lend support to extending aspects of the framework by including an additional enacting factor, withholding voice. Our findings indicate that all three enabling factors (support from supervisors/clinical leaders and hospital management for patient safety, and psychological safety) were inversely related to nurses’ withholding their voice. Moreover, nurses who reported withholding their voice less frequently, reported higher levels of overall patient safety in their unit. This may be because they perceive themselves as contributing to higher levels of patient safety by speaking up with their ideas and concerns.

Some limitations should be noted. Most importantly, we were unable to analyze data at the unit level because we lacked data on the usual units of study participants. To protect their confidentiality, we did not ask participants to identify their nursing unit, and thus we were not able to aggregate data to the unit level. This is particularly relevant to the assessment of psychological safety, which Bisbey et al. [2] had conceptualized as a group-level factor, as a collective perception that the group was safe for risk-taking. Therefore, even though we found that psychological safety measured at the individual level was highly predictive, future researchers should consider measuring this factor at the group level. It may be that psychological safety functions as both an individual- and group-level enabling factor. Other limitations include the use of cross-sectional data which preclude causal inferences, and the possibility of common method bias as all our measures were self-reported [30]. Finally, there is uncertainty in generalizing the findings of this study beyond populations with characteristics similar to the participants in our study: mostly female and working full time in medical/surgical units in tertiary hospitals in South Korea.

## Conclusion

Overall, this study contributes to an understanding of the processes or pathways by which aspects of safety culture may impact patient safety outcomes, and demonstrates the utility of the theoretical framework developed by Bisbey et al. [2]. The findings suggest that nurse assessments of the overall patient safety in their work settings are influenced by their enacting behaviours, which in turn are influenced by the enabling factors of safety culture. Both nurse leaders at the unit level and hospital administrators play an important role in developing a culture of safety culture and improving patient safety by creating environments that support nursing staff behaviours that lead more directly to positive safety outcomes.

## Data Availability

The datasets generated and/or analysed during the current study are not publicly available due to them containing information that could compromise research participant consent but are available from the corresponding author on reasonable request.
